# Supplementary material for: RNA-Seq and genetic diversity analysis of faba bean (Vicia faba L.) varieties in China
Source: PeerJ. 2023 Jan 10;11:e14259. doi: 10.7717/peerj.14259 (PMC9838209; doi:10.7717/peerj.14259)
Supplement: Supplemental Information 1 — Unified national number, variety name and source name of 226 faba bean varieties. [file peerj-11-14259-s001.doc]

TableS1 226 faba bean varieties derived from different provinces of China.

| Serial number | Unified national number | Variety name | Source place | Serial number | Unified national number | Variety name | Source place |
| --- | --- | --- | --- | --- | --- | --- | --- |
| 1 | H0000002 | Faba bean | Shanxi | 114 | H0001806 | Old faba bean | Shanxi |
| 2 | H0000006 | Damaya | Shanxi | 115 | H0001808 | Mi faba bean | Shanxi |
| 3 | H0000015 | Yang faba bean | Shanxi | 116 | H0001824 | green husk faba bean | Shanxi |
| 4 | H0000016 | Jia faba bean | Shanxi | 117 | H0001852 | Green faba bean | Shanxi |
| 5 | H0000039 | Baimaya | Shanxi | 118 | H0001854 | Kangle Niutaban | Shanxi |
| 6 | H0000090 | Chestnut beans | Inner Mongolia | 119 | H0001855 | Lintan faba bean | Gansu |
| 7 | H0000091 | Red faba bean | Inner Mongolia | 120 | H0001859 | Wenxian faba bean | Gansu |
| 8 | H0000095 | Local tree beans | Inner Mongolia | 121 | H0001860 | Wudu Yangyan bean | Gansu |
| 9 | H0000109 | Niutabian | Jiangsu | 122 | H0001862 | Tanchang faba bean | Gansu |
| 10 | H0000110 | Qingpi | Jiangsu | 123 | H0001863 | Minxian faba bean | Gansu |
| 11 | H0000113 | Niutabian | Zhejiang | 124 | H0001864 | Wushan faba bean | Gansu |
| 12 | H0000116 | Small seeds and long pods | Zhejiang | 125 | H0001865 | Zhangxian old soybean | Gansu |
| 13 | H0000117 | Cui wan Bai pi bean | Zhejiang | 126 | H0001866 | Lixian faba bean | Gansu |
| 14 | H0000120 | March yellow | Zhejiang | 127 | H0001867 | Qingshui Baijia bean | Gansu |
| 15 | H0000138 | Chuanhuang Small grain species | Zhejiang | 128 | H0001869 | Xihe faba bean | Gansu |
| 16 | H0000139 | Zaocanbai | Zhejiang | 129 | H0001871 | Yuzhong faba bean | Gansu |
| 17 | H0000152 | Qingpi dajiaoban | Hubei | 130 | H0001877 | Minqin faba bean | Gansu |
| 18 | H0007410 | Qinghai No.15 | Qinghai | 131 | H0001881 | Minle Hongshui faba Bean | Gansu |
| 19 | H0000158 | faba bean | Hubei | 132 | H0001884 | Lintao red broad bean | Gansu |
| 20 | H0000167 | White faba bean | Yunnan | 133 | H0001897 | Maya faba bean | Qinghai |
| 21 | H0000172 | Lashi bean | Yunnan | 134 | H0001900 | Red soybean | Qinghai |
| 22 | H0000184 | Green-skinned bean | Yunnan | 135 | H0001935 | Xipiga soybean | Qinghai |
| 23 | H0000189 | Baofeng faba bean | Yunnan | 136 | H0001937 | Donggouga soybean | Qinghai |
| 24 | H0000190 | Xinjie faba bean | Yunnan | 137 | H0001939 | Caojiabaoga soybean | Qinghai |
| 25 | H0000191 | Sunset Big faba Bean | Yunnan | 138 | H0001940 | Gamaya | Qinghai |
| 26 | H0000193 | Little red-skinned faba bean | Yunnan | 139 | H0001947 | Faba bean | Ningxia |
| 27 | H0000195 | Lianfeng faba bean | Yunnan | 140 | H0001953 | Faba bean | Ningxia |
| 28 | H0000200 | Majie faba bean | Yunnan | 141 | H0002517 | Chenghu No.1 | Sichuan |
| 29 | H0000201 | Sanchahe faba bean | Yunnan | 142 | H0002527 | Chenghu No.10 | Sichuan |
| 30 | H0000203 | Green leaves bean | Yunnan | 143 | H0002531 | Bing green husk faba bean | Jiangsu |
| 31 | H0000207 | Datongfu bean | Yunnan | 144 | H0002542 | Green faba bean | Ningxia |
| 32 | H0000216 | Zhongli bean | Yunnan | 145 | H0002621 | Purple faba bean | Ningxia |
| 33 | H0000218 | Fine faba bean | Yunnan | 146 | H0002622 | Faba bean | Ningxia |
| 34 | H0000242 | Eryuan bean | Yunnan | 147 | H0002692 | Faba bean | Xinjiang |
| 35 | H0000244 | Big faba bean | Yunnan | 148 | H0002693 | Faba bean | Xinjiang |
| 36 | H0000245 | Faba bean | Yunnan | 149 | H0002707 | Faba bean | Xinjiang |
| 37 | H0000247 | White faba bean | Yunnan | 150 | H0002709 | Qinghai No.1 | Gansu |
| 38 | H0000248 | Big red bean | Yunnan | 151 | H0003030 | Qinghai No.6 | Qinghai |
| 39 | H0000285 | Qingcan No.5 | Qinghai | 152 | H0003030 | Faba bean | Hebei |
| 40 | H0007373 | Qinghai No.13 | Qinghai | 153 | H0003109 | Faba bean | Hebei |
| 41 | H0005715 | Qinghai No.12 | Qinghai | 154 | H0003112 | Baimaya faba bean | Inner Mongolia |
| 42 | H0005714 | Qinghai No.11 | Qinghai | 155 | H0003126 | Maya faba bean | Inner Mongolia |
| 43 | H0000918 | soybean | Shanxi | 156 | H0003128 | Taixian green husk | Jiangsu |
| 44 | H0000930 | Damaya | Shanxi | 157 | H0003174 | Faba bean | Jiangsu |
| 45 | H0001019 | Jurong faba bean | Jiangsu | 158 | H0003186 | Faba bean | Jiangsu |
| 46 | H0001029 | Big white skin | Jiangsu | 159 | H0003191 | Kunshan green husk | Jiangsu |
| 47 | H0001030 | Niutabian | Jiangsu | 160 | H0003192 | Pukou faba bean | Jiangsu |
| 48 | H0004543 | Qinghai No.10 | Qinghai | 161 | H0003195 | Faba bean | Hunan |
| 49 | H0001033 | Haian white skin | Jiangsu | 162 | H0003210 | Faba bean | Hunan |
| 50 | H0001034 | Dantu Dali | Jiangsu | 163 | H0003305 | small faba bean | Guizhou |
| 51 | H0001035 | Dantu Xiaoli | Jiangsu | 164 | H0003333 | Tongmian faba bean | Guizhou |
| 52 | H0001037 | Jurong white skin | Jiangsu | 165 | H0003337 | Green skin and green heart beans | Yunnan |
| 53 | H0001039 | Nanguo bean | Jiangsu | 166 | H0003342 | Yanjiang bean | Yunnan |
| 54 | H0001075 | Yellow skin Tianjiqing | Zhejiang | 167 | H0003362 | Big faba bean | Yunnan |
| 55 | H0001079 | Xiangbai bean | Zhejiang | 168 | H0003370 | Dianzhong big white bean | Yunnan |
| 56 | H0001080 | Yellow skin Xiyeqing | Zhejiang | 169 | H0003375 | Small faba bean | Yunnan |
| 57 | H0001082 | native species | Zhejiang | 170 | H0003404 | Yangtian faba bean | Yunnan |
| 58 | H0001085 | Ningbochuan | Zhejiang | 171 | H0003415 | White faba bean | Yunnan |
| 59 | H0001086 | Zhongzi | Zhejiang | 172 | H0003430 | Maliao bean | Yunnan |
| 60 | H0001091 | Chihuai bean | Zhejiang | 173 | H0003431 | Yun bean 83-324 | Yunnan |
| 61 | H0001105 | Shimenqing | Zhejiang | 174 | H0004122 | Qinghai No.9 | Qinghai |
| 62 | H0001108 | Small faba bean | Zhejiang | 175 | H0003433 | 3Qinghai 3 | Qinghai |
| 63 | H0001109 | Qingtang green beans | Zhejiang | 176 | H0003761 | Faba bean | Jiangxi |
| 64 | H0001112 | Local March yellow | Zhejiang | 177 | H0003897 | Faba bean | Hunan |
| 65 | H0001114 | Kuobanqing | Zhejiang | 178 | H0003937 | Nazu faba bean | Sichuan |
| 66 | H0001290 | Small Buddha bean | Zhejiang | 179 | H0003944 | Luhua faba bean | Sichuan |
| 67 | H0001346 | Daqingpian | Anhui | 180 | H0003950 | Daikin native species | Sichuan |
| 68 | H0001381 | Highland barley broad bean | Hubei | 181 | H0004015 | small faba bean | Shanxi |
| 69 | H0001392 | Faba bean | Jiangxi | 182 | H0004033 | Kang faba bean | Shanxi |
| 70 | H0001402 | Faba bean | Jiangxi | 183 | H0004034 | Qinghai No.7 | Qinghai |
| 71 | H0001407 | Faba bean | Jiangxi | 184 | H0004288 | Baita green husk | Jiangsu |
| 72 | H0001425 | Big green husk faba bean | Hubei | 185 | H0004291 | Zhongzhuang small green husk | Jiangsu |
| 73 | H0001431 | Dake faba bean | Hubei | 186 | H0004297 | Xiaohuangshan green husk | Jiangsu |
| 74 | H0001432 | Xieban bean | Hubei | 187 | H0004299 | Yaowan green husk | Jiangsu |
| 75 | H0001433 | Xiaofanli faba bean | Hubei | 188 | H0004304 | Zhongzhuang white skin | Jiangsu |
| 76 | H0001438 | Faba bean | Hunan | 189 | H0004311 | Yaowan white skin | Jiangsu |
| 77 | H0001600 | Faba bean | Hunan | 190 | H0004355 | Faba bean | Anhui |
| 78 | H0001602 | Faba bean | Guangxi | 191 | H0004357 | Buddha bean | Anhui |
| 79 | H0001624 | Dabai faba bean | Sichuan | 192 | H0004360 | South slope small faba bean | Guangxi |
| 80 | H0001629 | Big faba bean | Sichuan | 193 | H0004363 | Puppy beans | Guangxi |
| 81 | H0001633 | Erban bean | Sichuan | 194 | H0004518 | Qingyi large faba bean | Yunnan |
| 82 | H0001635 | Dabai faba bean | Sichuan | 195 | H0004805 | Jinzhong bean | Yunnan |
| 83 | H0001637 | Erfanzao | Sichuan | 196 | H0004808 | Cunjiao bean | Yunnan |
| 84 | H0001645 | Erqing faba bean | Sichuan | 197 | H0004812 | Jinzhong bean | Yunnan |
| 85 | H0001652 | Qing faba bean | Sichuan | 198 | H0005047 | Ga soybean | Qinghai |
| 86 | H0001667 | Erbai faba bean | Sichuan | 199 | H0005075 | faba bean | Qinghai |
| 87 | H0001668 | Dabai faba bean | Sichuan | 200 | H0005088 | Chenqiao Baipi faba bean | Jiangsu |
| 88 | H0001669 | Tiejia faba bean | Sichuan | 201 | H0005089 | Nantong Sanbai | Jiangsu |
| 89 | H0001671 | Dabai faba bean | Sichuan | 202 | H0005091 | Tongcan No.5 | Jiangsu |
| 90 | H0001693 | Water faba bean | Sichuan | 203 | H0005094 | Su 89027 | Jiangsu |
| 91 | H0001701 | Green faba bean -1 | Sichuan | 204 | H0005277 | Beiyuanga faba bean | Gansu |
| 92 | H0001705 | Green faba bean | Sichuan | 205 | H0005320 | Pinliang Canxuan | Gansu |
| 93 | H0001706 | Red faba bean | Sichuan | 206 | H0005351 | Weiyuan Maya faba bean | Gansu |
| 94 | H0001718 | Mianhua faba bean | Guizhou | 207 | H0005366 | Ma's temple ditch faba bean | Gansu |
| 95 | H0001722 | Ximi faba bean | Guizhou | 208 | H0005383 | Kang maya xuan(Redskins) | Gansu |
| 96 | H0001724 | Big faba bean | Guizhou | 209 | H0005388 | Minxian Yangyan faba bean | Gansu |
| 97 | H0001731 | Er faba bean | Guizhou | 210 | H0005389 | Minhega bean126 | Gansu |
| 98 | H0001737 | Big faba bean | Guizhou | 211 | H0005680 | faba bean | Zhangjiakou |
| 99 | H0001758 | Ximi faba bean | Guizhou | 212 | H0005837 | faba bean | Chongqing |
| 100 | H0001760 | Dabai faba bean | Guizhou | 213 | H0005839 | faba bean | Chongqing |
| 101 | H0001761 | Shanbao White skin | Yunnan | 214 | H0005852 | Lincan No.2 | Gansu |
| 102 | H0001773 | Faba bean | Yunnan | 215 | H0005853 | Gaotai soybean | Gansu |
| 103 | H0001775 | Qinghe faba bean | Yunnan | 216 | H0005867 | faba bean | Chongqing |
| 104 | H0001776 | Yanhe faba bean | Yunnan | 217 | H0006305 | Guoyang daqinghua | Anhui |
| 105 | H0001777 | Huluchong bean | Yunnan | 218 | H0006306 | Hefei faba bean | Anhui |
| 106 | H0001780 | Cuoke bean | Yunnan | 219 | H0006309 | Daxian Fushou Local faba bean | Sichuan |
| 107 | H0001781 | Caoqing bean | Yunnan | 220 | H0006312 | Jinchuan local faba bean | Sichuan |
| 108 | H0001784 | Longzi Dabai bean | Yunnan | 221 | H0006313 | Emei faba bean | Sichuan |
| 109 | H0001791 | Jiangchuan green leaves bean | Yunnan | 222 | H0003027 | Qinghai 1 hao | Qinghai |
| 110 | H0001796 | Semi-soybean | Yunnan | 223 | H0003028 | Qinghai No.2 | Qinghai |
| 111 | H0001797 | Nagu faba bean | Yunnan | 224 | H0000284 | Qinghai No.3 | Qinghai |
| 112 | H0001802 | faba bean | Yunnan | 225 | H0003029 | Qinghai No.4 | Qinghai |
| 113 | H0001805 | faba bean | Shanxi | 226 | H0000285 | Qinghai No.5 | Qinghai |
