# Supplementary material for: RNA-Seq and genetic diversity analysis of faba bean (Vicia faba L.) varieties in China
Source: PeerJ. 2023 Jan 10;11:e14259. doi: 10.7717/peerj.14259 (PMC9838209; doi:10.7717/peerj.14259)
Supplement: Supplemental Information 3 [file peerj-11-14259-s003.docx]

**Table S3 Overview of the RNA-seq data**

| Sample | Raw Reads | Clean Reads | Clean Bases | GC Content (%) | Clean Reads Q20 | Clean Reads Q30 | Clean Reads Ratio | Total Mapping | Uniquely Mapping (%) |
| --- | --- | --- | --- | --- | --- | --- | --- | --- | --- |
|  | (M) | (M) | (Gb) |  | (%) | (%) | (%) | (%) |  |
| Qinghai11-1 | 60.3 | 52.21 | 7.88 | 39.41 | 98.22 | 94.82 | 86.59 | 39.79 | 11.53 |
| Qinghai11-2 | 58.43 | 49.92 | 7.54 | 39.37 | 98.25 | 94.92 | 85.44 | 40.02 | 11.67 |
| Qinghai11-3 | 55.37 | 47.37 | 7.15 | 39.65 | 98.2 | 94.77 | 85.55 | 39.8 | 11.84 |
| Qingcan16-1 | 57.63 | 47.98 | 7.25 | 39.56 | 98.21 | 94.82 | 83.26 | 39.19 | 12.57 |
| Qingcan16-2 | 58.79 | 50.77 | 7.67 | 39.69 | 98.2 | 94.78 | 86.37 | 39.5 | 12.46 |
| Qingcan16-3 | 69.91 | 60.34 | 9.11 | 39.64 | 98.25 | 94.91 | 86.3 | 39.62 | 12.49 |
| Qingcan18-1 | 58.14 | 50.25 | 7.59 | 39.55 | 98.22 | 94.84 | 86.42 | 40.37 | 12.77 |
| Qingcan18-2 | 57.95 | 49.93 | 7.54 | 39.48 | 98.22 | 94.83 | 86.16 | 40.82 | 12.89 |
| Qingcan18-3 | 57.08 | 48.92 | 7.39 | 39.42 | 98.22 | 94.85 | 85.7 | 40.42 | 12.51 |
| YL01-1 | 56.94 | 48.82 | 7.37 | 39.61 | 98.21 | 94.79 | 85.73 | 38.94 | 11.7 |
| YL01-2 | 62.18 | 53.22 | 8.04 | 39.36 | 98.21 | 94.81 | 85.58 | 39.71 | 11.75 |
| YL01-3 | 61.31 | 52.3 | 7.9 | 39.53 | 98.23 | 94.87 | 85.3 | 39.72 | 11.64 |

Notes: _1,_2 and _3 represent the three biological replicates. M represents million. GC Content represents the percentage of guanine and cytosine in the clean reads. Q30 represents the percentage of nucleotides with a quality value ≥ 30.
