# Supplementary material for: RNA-Seq and genetic diversity analysis of faba bean (Vicia faba L.) varieties in China
Source: PeerJ. 2023 Jan 10;11:e14259. doi: 10.7717/peerj.14259 (PMC9838209; doi:10.7717/peerj.14259)
Supplement: Supplemental Information 4 — Analysis of unigenes [file peerj-11-14259-s004.docx]

Table S4 Quality index of unigenes

| Samples | Total Number (bp) | Total Length  (bp) | Mean Length  (bp) | N50 | N70 | N90 | GC(%) |
| --- | --- | --- | --- | --- | --- | --- | --- |
| Qinghai11-1 | 58,392 | 63389970 | 1085 | 1606 | 1092 | 512 | 39.41 |
| Qinghai11-2 | 57,279 | 62199850 | 1085 | 1581 | 1087 | 522 | 39.37 |
| Qinghai11-3 | 51,963 | 52570595 | 1011 | 1483 | 1006 | 466 | 39.65 |
| Qingcan16-1 | 54,883 | 56234601 | 1024 | 1505 | 1022 | 480 | 39.56 |
| Qingcan16-2 | 53,008 | 53190699 | 1003 | 1483 | 998 | 463 | 39.69 |
| Qingcan16-3 | 52,992 | 54094105 | 1020 | 1495 | 1015 | 476 | 39.64 |
| Qingcan18-1 | 55,274 | 58266838 | 1054 | 1547 | 1057 | 496 | 39.55 |
| Qingcan18-2 | 54,821 | 57437733 | 1047 | 1524 | 1046 | 500 | 39.48 |
| Qingcan18-3 | 56,997 | 61512065 | 1079 | 1579 | 1084 | 517 | 39.42 |
| YL01-1 | 53,121 | 54082090 | 1018 | 1486 | 1013 | 481 | 39.61 |
| YL01-2 | 59,951 | 66897250 | 1115 | 1634 | 1125 | 539 | 39.36 |
| YL01-3 | 55,140 | 58789376 | 1066 | 1569 | 1066 | 504 | 39.53 |
| All-Unigene | 133,487 | 178152541 | 1334 | 1858 | 1330 | 708 | 38.77 |

Notes: _1,_2 and _3 represent the three biological replicates.
