# Supplementary material for: RNA-Seq and genetic diversity analysis of faba bean (Vicia faba L.) varieties in China
Source: PeerJ. 2023 Jan 10;11:e14259. doi: 10.7717/peerj.14259 (PMC9838209; doi:10.7717/peerj.14259)
Supplement: Supplemental Information 6 — Sequences of developed SSR markers. SSR motif and repeats were analyzed with Microsatellite identification tool and the primers were designed using Primer 3.0. [file peerj-11-14259-s006.docx]

Table S6 Sequences of 103 polymorphic SSR markers used for genetic diversity analysis

| NO | SSR name | SSR | FORWARD PRIMER1 (5'-3') | Tm | REVERSE PRIMER1 (5'-3') | Tm |
| --- | --- | --- | --- | --- | --- | --- |
| 1 | T_DN32469 | (TAA)8 | ACGGTTCCTTCTGGTTGTTG | 60 | TTTTTCGTAACGCCATCACA | 60 |
| 2 | T_DN24459 | (CTC)5 | CCGCCATTCGGTGTATTTAG | 60 | GCCAAAAACTGCAACCATTT | 60 |
| 3 | T_DN27482 | (GAA)5 | CAGCTCTCAAACCGTTCTCC | 60 | TTCCCAAACACTTCACTCCC | 60 |
| 4 | T_DN22376 | (TA)8 | TGATTCCCACACAAACACTTG | 59 | AACGGTTGTCGAGGTTTCAC | 60 |
| 5 | T_DN29279 | (TGA)5 | CCCTAGTTCACCACCAGGAA | 60 | GTGGCTTTGTACTTGGGGAA | 60 |
| 6 | T_DN1835 | (GA)7 | TACCTGGGCTTGGCTTTATG | 60 | TTCTGTTAATGTTAGAATATGGACCC | 59 |
| 7 | T_DN33317 | (TC)8 | GATCATGCAAAAAGCAAGCA | 60 | GAGAAAGTGGGTAGCTGGGA | 59 |
| 8 | T_DN24385 | (GTT)6 | TAGCAGCGGAGGAGTAGGAA | 60 | AGAAAAGCACCTCTACCGCA | 60 |
| 9 | T_DN26689 | (ACA)5 | AACACAACCACAAACCAGCA | 60 | TACCCCAGTCATTGCAACTT | 58 |
| 10 | T_DN23895 | (TGA)6 | GCATCCTCTTCAACCAAAGC | 60 | TTGTCCAATCAATAACCGCA | 60 |
| 11 | T_DN31712 | (CAT)5 | TTCACCTCCAATCTCCATCA | 59 | TGCTTTGATGGCATTTCAAC | 60 |
| 12 | T_DN20824 | (GCG)5 | CCGTAGTTGACGCCGATATT | 60 | CTTCTATCAAATTCCCCGCA | 60 |
| 13 | T_DN28915 | (CAT)7 | TGAAAACTCTTCCTCTCGGC | 60 | TGTGATGTTGATCATTGCCC | 60 |
| 14 | T_DN32959 | (AG)7 | CTCGTTTTCTCCGATCCAAC | 60 | TTTGAGAAATACAAAATCAGTTGC | 58 |
| 15 | T_DN24924 | (CT)10 | TCATGACAGTAACAACTGTAAAGAAGA | 59 | TGAAGTGAGAGAGTCGAGGGA | 60 |
| 16 | T_DN27054 | (TTG)6 | TCATCCCTTGTCTCTCACCA | 59 | TCCATGGAAAACCAAAACAA | 59 |
| 17 | T_DN27054 | (TTG)8 | TTTTGGTTTTCCATGGAGTTG | 60 | ACCCAAATGAAATCAGTGGC | 60 |
| 18 | T_DN32461 | (AGA)5 | AAGACCATGAAGGCATGAGG | 60 | TCATCAATGGCTTCCACAAA | 60 |
| 19 | T_DN32430 | (AAC)5 | AATCGGTGAAAACGAAAACG | 60 | AAAAGCCGGCACTATCACAG | 60 |
| 20 | T_DN32302 | (AG)10 | GAGAGATTTTGCTTCCGTCG | 60 | TAACAAACTCGAGGCAGGCT | 60 |
| 21 | T_DN23241 | (AG)21 | GCAACTTGTTCAGCGTTTGA | 60 | CCGAAGTAAAAGAGATTGAGCC | 59 |
| 22 | T_DN29012 | (GAG)5 | TGTGAAGTCCGTGACACCAT | 60 | CTTTCCATCGCTTCACCTGT | 60 |
| 23 | T_DN20702 | (AT)14 | TCATTTCAACGTACCAATGGAT | 59 | ATTTGGAGGGTTTTTGGGAC | 60 |
| 24 | T_DN31208 | (TGA)5 | AGTTCCCTCTTTGGTCCCAT | 60 | GAGAGTGAAGGCGTGTGTTG | 59 |
| 25 | T_DN24534 | (TG)10 | TGCACAGTCAAAGCATGTCA | 60 | AATCACAAGTGTCGGTGCAG | 60 |
| 26 | T_DN7799 | (AC)6 | CCATCAACAACCTCACATGC | 60 | AGGCTCTGATACCACGCTGT | 60 |
| 27 | T_DN20827 | (AAC)5 | CATCCAAACCATACCCAATCA | 60 | TGAAGTTAGCAATGGGGCTC | 60 |
| 28 | T_DN23877 | (GAA)5 | GCGGAGGTTGAAATTTGTGT | 60 | CCCTCACTTTCCCATCCTTT | 60 |
| 29 | T_DN27103 | (AG)11 | TGTTGCCAGATGAGACAAGG | 60 | CCTCTCTGCAAGCTGCTTTT | 60 |
| 30 | T_DN19607 | (AG)6 | GAAAGGGACAACAAGAGAAGTCA | 60 | GATTGTGAATTGGGTGCTCA | 60 |
| 31 | T_DN26771 | (ATC)6 | TTGAGACACTTCAGCTAGTATCACG | 60 | GAACCGACAATTGATCAGGAA | 60 |
| 32 | T_DN27978 | (CCG)5 | CTCCCAACTTGCAAATCCTC | 60 | TAATAGCTGCCATGAGCGAA | 60 |
| 33 | T_DN25216 | (AAC)5 | TGAAAGCAATTGCGTACGAG | 60 | TCACCATCTTCCGCAATACA | 60 |
| 34 | T_DN30193 | (TCT)7 | ACTCCCCTTGCTCCTCATCT | 60 | CTCGAGCCACAACAACTCAA | 60 |
| 35 | T_DN9246 | (GTT)6 | GCTAGTGGACCTCCCATTGA | 60 | TCCAATGCAAACTCTCCAAA | 59 |
| 36 | T_DN19478 | (A)10 | GCAAACGAAGATAAACCACAAA | 59 | TTTGTGAAAGTGTGGGTGTGA | 60 |
| 37 | T_DN34083 | (TTA)6 | ATCGTCGTCGTTTCTACGCT | 60 | TAGAATTTCCGAAAATGGCG | 60 |
| 38 | T_DN9805 | (GT)7 | GCCTCGTTCTCGTATGTTTCA | 60 | GTCACCACAGTCCTAGGGGA | 60 |
| 39 | T_DN30600 | (AC)9 | AGCCAATTTGACCCATCATT | 59 | TCCTCCCTTAGTGGCTCTTG | 59 |
| 40 | T_DN9218 | (AC)6 | CCAGAAAACATCCTCAACCA | 59 | CCGAACCTAAGCCACGTTAC | 60 |
| 41 | T_DN23793 | (TTG)5 | TGATCGGCTTTGACACAGAG | 60 | GGGCTTGCTTCCTAAAGAGG | 60 |
| 42 | T_DN23793 | (GTC)5 | AACGCGTGATGCTTCTTCTT | 60 | TCCCACATTGCACTTCAAAA | 60 |
| 43 | T_DN30269 | (TTG)7 | GGTTCGGAGTTAGGTGTGGA | 60 | AGCCATTCCATTCCCTCTTT | 60 |
| 44 | T_DN26655 | (AG)6 | GGCTTGTACCGATCGTGTTT | 60 | CCCTTCCCTCAACCTCATTC | 61 |
| 45 | T_DN18457 | (T)10 | TGGCAATGGAAAGTGAAAGA | 59 | TCCAAGGGTGGAAGTTTTTCT | 60 |
| 46 | T_DN26075 | (ATC)7 | TTCACACACCCAACAAACAAA | 60 | GAATGGTGGTGGAGGAAGAA | 60 |
| 47 | T_DN27071 | (CAA)6 | GATTCTCGCAGTTTCGGTTC | 60 | TGGGGTTGTTGTGTTGTTGT | 60 |
| 48 | T_DN29286 | (A)10 | CGGGATAATTAGCACAAGCAA | 60 | AGGGGCCTCTCTATCCCATA | 60 |
| 49 | T_DN32727 | (CATTA)5 | TGCCAAATTAACCTCATTCCTT | 60 | ATACGTGTGTGCCTGCAGAT | 59 |
| 50 | T_DN29213 | (AAC)5 | TCATGATCGTGCCTTAGCAG | 60 | TTTGATCAATGTAGTAGCCGATTC | 60 |
| 51 | T_DN33153 | (CT)7 | TCAAGGCTCTCTGCCATTTT | 60 | ATGCATCCGGTTATGGTGAT | 60 |
| 52 | T_DN32381 | (TC)18 | TGAGGGACCATGGCAGTTAT | 60 | GCCACCACTGTGGAGTTCAT | 61 |
| 53 | T_DN33688 | (CT)15 | GTTTCCCAACCCTCAATCAA | 60 | CCTTGGTGCATAATAGGGGA | 60 |
| 54 | T_DN24007 | (AG)9 | ACCCATGACGGTGAATTGAT | 60 | AATCGAGCCAAAATAGCACG | 60 |
| 55 | T_DN25422 | (CT)6 | GCCAACTCATACGACCGATT | 60 | TCTTGGTGATTCCTCTGCAA | 59 |
| 56 | T_DN34120 | (GAA)6 | AAAGAAGCATGAAACTGGGG | 59 | CCTTGGCAAGCTGAAAGAAG | 60 |
| 57 | T_DN29810 | (GTG)5 | GTGACTTGAGCGAATGCAAA | 60 | TGTTAACCTCAATGCCACCA | 60 |
| 58 | T_DN30324 | (TC)7 | TTGGGGATGACAGTTTGAAT | 58 | GTTGCAGCGATGAAACAGAA | 60 |
| 59 | T_DN26787 | (CCA)5 | CATAACCTTCACCATCGCCT | 60 | TTCATCGTCACCTAACAGCG | 60 |
| 60 | T_DN32610 | (TGG)5 | AGCTTTCAGGGGGTTTGTTT | 60 | GGCACAACCTTGTAATGCCT | 60 |
| 61 | T_DN16768 | (AT)6 | GTCTCAGGGAATTGATCGGA | 60 | TTATTACCTTCGAAAGCACGC | 59 |
| 62 | T_DN26565 | (A)13 | AAGCTTAGAAGAAATTCAGCAAAAA | 59 | TCCACCACTACCAAACCCTC | 60 |
| 63 | T_DN31063 | (AT)9 | TCCATCAAACTTGTGCATGA | 59 | TTCAGGCAAGTTAAGATTCTTGG | 60 |
| 64 | T_DN22109 | (A)11 | CCATAGAAGAAACTAATTTGCCC | 58 | AGTTCGCGATACATCCCAAG | 60 |
| 65 | T_DN28291 | (TC)6 | CATTGTGCATGTGCAGTAACA | 59 | TCTGAATCGCAAACAAGCTC | 59 |
| 66 | T_DN23988 | (TTTC)6 | AAGGGGAAAAGGAAGGTGTG | 60 | CCTTAGGCATCACAGCACAA | 60 |
| 67 | T_DN24104 | (CT)7 | AAGCACCCTTATTCCATCAAA | 59 | GTTGGATCCGGGTTTTCTCT | 60 |
| 68 | T_DN29493 | (A)10 | TCGTGTTCATTCTGCCTGAG | 60 | TACTGCGCACATCCTAGTGG | 60 |
| 69 | T_DN25939 | (AG)8 | TGTTTGGAAAAAGAAGGCACA | 61 | CCAAAACAAAAACCATTCCG | 60 |
| 70 | T_DN34009 | (GATT)6 | GGAATTGAAGCCCTAAACCC | 60 | ACTTGAACCCCCTTTTTGCT | 60 |
| 71 | T_DN25127 | (GA)9 | CCGCATTGAAGTTTTTAGCC | 60 | CATTCACCCCACGTTTCTCT | 60 |
| 72 | T_DN33367 | (TG)6 | CTGGTCGTCTGAGTAAGGGG | 60 | CCACACCCAACCAAAATCTC | 60 |
| 73 | T_DN28175 | (TTG)5 | ATGATGGTGGTGGTGAGGTT | 60 | ATATCAACCCGATCCTTCCC | 60 |
| 74 | T_DN26387 | (ATC)5 | AAAAATCCCCCTGCATCTTC | 60 | AGGTGTTGCTCTGAATTGGC | 60 |
| 75 | T_DN25712 | (CTT)5 | TGGTCATCTTCTTCAGCGTG | 60 | AAGAAGAAAAAGAAGCCCGC | 60 |
| 76 | T_DN29236 | (A)11 | AAATTAGCATATGGAGCATAGTGAA | 58 | GCATTGATGCAAAAGGGAGT | 60 |
| 77 | T_DN30971 | (GA)9 | TGTTGACGGTGGATGGAGTA | 60 | TGGATTCTCAACGTGACTCG | 60 |
| 78 | T_DN28375 | (GA)8 | ACGCAAAGAAAGAACCGAGA | 60 | TTTCCACCACTCTCCTCCAC | 60 |
| 79 | T_DN23472 | (AG)8 | GGTTTTTGTTTTGCTGCGAT | 60 | ACCAACGAATCATTCTGCAA | 59 |
| 80 | T_DN28326 | (AG)6 | CTTGCGCATGTTGAAGAAAA | 60 | TGCAGCCACAATTCAACACT | 60 |
| 81 | T_DN24259 | (A)10 | AGCCCCTCCTTTTCCAAATA | 60 | TGGTCTCAAATGTGGTGGAA | 60 |
| 82 | T_DN30467 | (TC)10 | CACCACCAACTGCACCTTC | 60 | TCTCGCTTACGATTCTGGTG | 59 |
| 83 | T_DN30467 | (AGA)5 | CACTGCAACCTTCACCTTCA | 60 | TGCTCAGGTTCGCCTTATCT | 60 |
| 84 | T_DN12372 | (T)10 | AGGCATCAAAGAGGAACTGC | 59 | GCACATGGGTTAGCAGGATT | 60 |
| 85 | T_DN25819 | (ATG)5 | GTTGTAAGGTCAGGGGCTCA | 60 | AAGGTTTTTGCAACACTTCCA | 60 |
| 86 | T_DN28542 | (A)11 | TGGATTCCATCATATTCCGC | 61 | TGACTATGGGTGTGGGAAAA | 59 |
| 87 | T_DN17703 | (AG)7 | TTGGAAGGAAGTGAACCACC | 60 | TTGTTCAGGTGATGCAAGAGA | 59 |
| 88 | T_DN24002 | (TC)12 | CCCTCTCTCTTTTGCTTTCG | 59 | GAGACTGAGCGAGCCATACC | 60 |
| 89 | T_DN30331 | (GAA)5 | CTAAGGCAAGAGCAACGGTC | 60 | CCCAAAGACAATCCAGTCCT | 59 |
| 90 | T_DN23145 | (GAA)5 | CCCGAAAAATTTCGAGAAGA | 59 | TGACGGATCTAGGGTTCCAG | 60 |
| 91 | T_DN33887 | (CAA)6 | CGAACAGTACCGGAACAAAAA | 60 | CACCACCATCCTTGCTCTTT | 60 |
| 92 | T_DN25920 | (A)11 | GCCTACTTCTGAAGGCATGG | 60 | GGGCTTCCTCCTCAGTTTTC | 60 |
| 93 | T_DN30840 | (TGT)6 | AGTGTGAGGGTGGTGGTAGC | 60 | GCCAAGTGGTGAACAAGGTT | 60 |
| 94 | T_DN24924 | (AG)8 | GCAGCAAGAACGACCATTTT | 60 | ATGCATGAGTTTGTTTTGCG | 60 |
| 95 | T_DN20915 | (TCA)5 | TCAGTTGGAGGCATAGGAGG | 60 | TCAAACTTCATGGCTTTCCC | 60 |
| 96 | T_DN29632 | (T)11 | GTCGATGTTTCTTTTGCAGC | 59 | TTTGAATTCCTTTTCTTTTGTTTG | 59 |
| 97 | T_DN15279 | (AT)8 | TCCGCCATGAATAATTTGGT | 60 | GCGAGCTAACGCTCAGAATC | 60 |
| 98 | T_DN31937 | (CT)9 | AGATCAAGAACTCACCGCAAA | 60 | GATTCGCTGATGAAGGTGGT | 60 |
| 99 | T_DN22251 | (A)10 | CATGTCAATCCACAATCTCTTCA | 60 | GTGTCATTGAGTGTCGGTGC | 60 |
| 100 | T_DN32482 | (ACA)5 | TCCCCCAAAGCAATCAATAG | 60 | TTGTCCGGAGAGTTGTTTTTG | 60 |
| 101 | T_DN32707 | (ACA)6 | GTGAGAATGGGGGATGAAAA | 60 | ATCGCCGGCTTAGTATGAAA | 60 |
| 102 | T_DN34099 | (A)12 | TGTGAAAATAGGGGCCTCAG | 60 | AAGAGGCAACCCTAATCCGT | 60 |
| 103 | T_DN29047 | (ATA)5 | TTTGCACCACTTTTATGCCA | 60 | AGAAGGAAAACCTGGACGGT | 60 |
